# 2494 Selectives: Implementing self-directed collaborative selectives as part of a curriculum for pre-health care professional students

**DOI:** 10.1017/cts.2018.225

**Published:** 2018-11-21

**Authors:** Leonor Corsino, Stephanie A. Freel, Melanie Bonner, Joan Wilson, Christie McCray, Maureen Cullins, Linda S. Lee, Kathryn M. Andolsek

**Affiliations:** 1 Duke University; 2 Clinical Research Education & Outreach, Duke Office of Clinical Research (DOCR); 3 Professor in Psychiatry and Behavioral Sciences, Duke School of Medicine; 4 Research Pro

## Abstract

OBJECTIVES/SPECIFIC AIMS: To provide students an opportunity to select health care-oriented course work that reflects both their interests and the increasingly diverse spectrum of health professions education and health care careers. To increase the opportunity for students to enter professional schools and health care professions with enhanced engagement and experience. METHODS/STUDY POPULATION: The 4-credit elective (Selective) curriculum is a component of the 38 credit Duke School of Medicine Master of Science in Biomedical Sciences (MBS) program which is completed over 10.5 months. Students work closely with their advisors to choose activities that reflect their interests. Selectives are offered by an array of schools, institutes, and programs within Duke University, including: the School of Medicine, School of Law, Global Health Institute, Bioethics and Science Policy Master Program, Clinical Research Training Program, Center for Documentary Studies, and Medical Informatics. Students may also pursue directed studies in areas such as health policy, or an inter-professional trip to Honduras. In addition to the course-based Selectives, three research practicum options are offered: Community Engagement, Clinical Research (Duke Office of Clinical Research), and a self-selected mentored research experience. Finally, the MBS program offers 2 in-house specific Selectives: Fundamentals of Learning: Theory and Practice, and Planning for Health Professions Education. RESULTS/ANTICIPATED RESULTS: The MBS program accepted its first cohort of students in June 2015. Two cohorts have graduated and the third has begun (n=30, 2016; n=42, 2017; n=43 enrolled, 2018). Our students come from diverse background with a third from populations historically underrepresented in STEM due to race/ethnicity, and another third underrepresented due to other factors such as low socioeconomic status, first generation to college, LGBQT, and those from rural and immigrant communities. Thus far, Selective distribution has been: Clinical research practicum (7, 2016; 14, 2017; 9, 2018); Mentored research practicum (2, 2016; 1, 2017); Community engagement practicum (7, 2016; 4, 2017; 5, 2018); Planning for health professions educations (14, 2016; 32, 2017; 33, 2018), Fundamentals of learning: Theory and Practice (7, 2016; 17, 2017; 18, 2018); documentary film (1, 2016); inter-professional trip to Honduras (2, 2016, 2, 2017). Since the implementation of the curriculum, at least 53 of 70 students who have applied (76%) were admitted to health professions or other graduate schools despite having lower initial MCAT and undergraduate GPAs in aggregate than the average of students who matriculate to allopathic medical school programs: 41 to medical schools, 3 to dental school, 2 each to osteopathic and physician assistant schools and 1 each to physical therapy, business school and law school. Eighteen of the 2016 graduates, and 21 of the 2017 graduates work in research for their gap year following graduation, the majority being employed in our institution’s research programs providing a pipeline of trained research assistants and coordinators. DISCUSSION/SIGNIFICANCE OF IMPACT: Lessons learned by implementing our curriculum include the following: (1) students are eager to explore different areas of health care; (2) collaboration across schools, centers, departments, institutes, and offices increases our ability to identify common areas of interest; (3) implementing a diverse curriculum can be challenging due to the need for significant organization and planning; (4) the diversity of courses can be a source of confusion when there is a lack of standardization in learner expectations; (5) continued collaboration across, schools, centers, institutes programs, health professions and sections requires a significant amount of time and expertise. However, our programs demonstrate significant positive impacts both on students and at the institutional level. Our program shows that a diverse curriculum leads to a high number of students engaged in pursuing and successfully continuing a health profession education. Institutional benefits include a robust pipeline for a diverse research workforce.

